# *Akkermansia muciniphila*: new insights into resistance to gastrointestinal stress, adhesion, and protein interaction with human mucins through optimised *in vitro* trials and bioinformatics tools

**DOI:** 10.3389/fmicb.2024.1462220

**Published:** 2024-11-05

**Authors:** Franca Vergalito, Diletta Bagnoli, Lucia Maiuro, Gianfranco Pannella, Valentino Palombo, Bruno Testa, Francesca Coppola, Roberto M. A. Di Marco, Patrizio Tremonte, Silvia J. Lombardi, Massimo Iorizzo, Raffaele Coppola, Mariantonietta Succi

**Affiliations:** ^1^Department of Agricultural, Environmental and Food Sciences, University of Molise, Campobasso, Italy; ^2^Department of Science and Technology for Sustainable Development and One Health, University Campus Bio-Medico of Rome, Rome, Italy; ^3^Italian National Research Council (CNR), Institute of Food Sciences (ISA), Avellino, Italy; ^4^Department of Drug and Health Sciences, University of Catania, Catania, Italy

**Keywords:** *Akkermansia muciniphila*, *Lacticaseibacillus rhamnosus* GG, simulated gastrointestinal transit, adhesion, Caco2, HT-29, HT29-MTX, potential probiotic

## Abstract

According to the FAO/WHO guidelines, selection of probiotics requires the assessment of survival under gastrointestinal stress and adhesion to human epithelial cells. These attributes were evaluated on *Akkermansia muciniphila* ATCC BAA-835 simulating the gastrointestinal transit (GIT) immediately followed by adhesion to human intestinal cell lines (CaCo2, HT-29, and HT-29-MTX) as an alternative approach to *in vitro* methods performed with fresh cells in each trial. The survival rate after GIT, as determined by plate counts and fluorescent probes, was significantly higher for *A. muciniphila* (about 8 Log CFU/mL) than for the probiotic *Lacticaseibacillus rhamnosus* GG ATCC 53103 (about 3 Log CFU/mL). The use of Live/Dead assay highlighted that *A. muciniphila* forms cell aggregates in the gastric phase as protective mechanism, explaining its high viability in the intestine. The rate of adhesion to human cell lines was always lower for strains tested after simulated GIT than for strains that did not undergo simulated GIT. *Akkermansia muciniphila* exhibited significantly higher adhesion than *Lbs. rhamnosus* GG, particularly to the mucus-secreting HT-29-MTX cells across a range of concentrations (2–8 Log CFU/mL). Finally, the bioinformatic analysis of *A. muciniphila* proteome confirmed the Amuc_1434 as a potential factor in binding to the human MUC2 protein.

## 1 Introduction

*Akkermansia muciniphila* is a gram-negative, anaerobic, oval-shaped bacterium belonging to the *phylum* Verrucomicrobia (Cozzolino et al., 2020). It is considered a normal intestinal symbiont in the course of life. This bacterium colonises the human gut permanently within a year after birth, thanks to transfer through the mother’s milk (Collado et al., 2012). Its abundance in the gut reaches high levels in healthy adults, representing approximately 1–3% of the total microbiota, and then gradually decreases in the elderly (Collado et al., 2007; Derrien et al., 2008; Zhang et al., 2019). The strain ATCC BAA-835 was isolated in 2004 by Derrien et al. from a faecal sample of a healthy man (Derrien et al., 2004) and nowadays it is one of the most studied human intestinal colonisers due to its intriguing beneficial features. *Akkermansia muciniphila* can use mucin as carbon and nitrogen source, which gives it a competitive advantage during nutrient deficiencies in the gut (Van Passel et al., 2011; Derrien et al., 2017; Karamzin et al., 2021). Several studies showed that *A. muciniphila* is abundant in biopsies of healthy subjects and reduced in those from patients with inflammatory bowel disease, hypertension, obesity, atopic diseases, and autism (Png et al., 2010; Derrien et al., 2011; Wang et al., 2011; Candela et al., 2012; Dao et al., 2016). Therefore, the presence of this bacterium is considered as a significant biomarker of intestinal homeostasis and host physiology (Hansen et al., 2012; Le Chatelier et al., 2013). Such positive actions in humans can be attributed to its ability to produce metabolites, compete with other dangerous bacteria, and maintain the balance of immunity and integrity of the intestinal barrier (Everard et al., 2013; Li et al., 2017). Thanks to its mucolytic activity, *A. muciniphila* could partially reduce the severity of colitis (Inaba et al., 2023), and several studies assume its involvement in mucus production with positive effects on a variety of chronic pathological states (Routy et al., 2018). These aspects are however still debated, since recent studies showed that the over-colonisation of *A. muciniphila* in the gut, explored in mouse model, reduces the thickness of the intestinal mucus layer and damages the intestinal barrier, with negative effects that would eventually aggravate the development of colitis and colorectal cancer (Qu et al., 2023). These findings suggest that the mechanisms underlying the different effects of *A. muciniphila* on the intestinal, inflammatory and immune systems are still difficult to understand and involve different factors which deserve further investigation.

The use of the strain ATCC BAA-835 as a beneficial bacterium is currently restricted in pasteurised form for a target population of adults, excluding pregnant and lactating women, while its use as live probiotic is still under investigation [EFSA Panel on Nutrition, Novel Foods and Food Allergens (NDA), 2021]. In this context, the current guidelines indicate various criteria for evaluating *in vitro* and/or *in vivo* the efficacy of candidate probiotic organisms (Byakika et al., 2019). Among the major criteria to characterise potential probiotic bacteria, there are the survival to gastrointestinal transit (GIT) simulation and the adherence to human epithelial cells (FAO/WHO, 2020).

Once ingested, probiotics pass through the GI tract, from the mouth through the stomach to the small intestine and colon. Probiotics must tolerate oral enzymes, in particular lysozyme and resist the antimicrobial elements present in the stomach (low pH, acidic gastric fluid and pepsin) and intestine (pancreatin and bile salts) in order to temporarily persist in this environment and exert their health-promoting effects. In this field, several studies have evaluated the resistance of probiotic strains to gastric acidity and biliary toxicity, as well as their ability to colonise the GI system and produce antimicrobial compounds and modify immune responses (Succi et al., 2017; Galdeano et al., 2019; Letizia et al., 2022).

Probiotic adhesion relies on surface chemistry and membrane interactions, crucial for effective microbe-cell binding and intestinal colonisation (Boonaert and Rouxhet, 2000; Duary et al., 2011). In this regard, different human intestinal cell lines, such as CaCo2, HT-29 and HT-29-MTX, have been used to identify and subsequently evaluate potentially probiotic microorganisms based on their adhesion properties (Mantzourani et al., 2019; Rocha-Mendoza et al., 2020).

To the best of our knowledge, no information is available concerning the influence of GIT simulation on the subsequent adhesion capacity of probiotic strains. Assays simulating GIT and the adhesion ability are, in fact, often performed in two distinct trials, each conducted by using bacterial cultures grown in fresh media and optimal conditions (De Palencia et al., 2008; Jensen et al., 2012; Coelho-Rocha et al., 2023), without considering the impact that GI stress can have on adhesion. For this reason, in this work, for the first time, the two tests were performed consecutively on *A. muciniphila* strain ATCC BAA-835, using *Lacticaseibacillus rhamnosus* GG as a comparison. The latter actually represents one of the most widely studied probiotic bacteria (Capurso, 2019; Leser and Baker, 2024), marketed worldwide as a probiotic and still widely used in comparative studies concerning safety and performances of probiotic candidates. We believe that the execution of the two tests in sequence is a viable alternative to conducting them individually, as an adhesion assay with bacteria that have previously undergone GI stress represents a condition more similar to what occurs *in vivo*.

## 2 Materials and methods

### 2.1 Bacterial strains and culture preparation

Bacterial strains used in this study were *Akkermansia muciniphila* ATCC BAA-835 (ATCC, Valio Ltd., Helsinki, Finland) and *Lacticaseibacillus rhamnosus* GG ATCC 53103 (ATCC, Valio Ltd., Helsinki, Finland), the latter used for comparative purposes. Strains, stored at −80°C in glycerol (Succi et al., 2017), were propagated at 37°C for 48–72 h in anaerobiosis (Gas Pack AnaeroTM, Oxoid™), using as growth substrates de Man, Rogosa and Sharpe (MRS) broth (Oxoid, Milan, Italy) for *Lbs. rhamnosus* GG ATCC 53103 and BHI (Oxoid, Milan, Italy) for *A. muciniphila* ATCC BAA-835.

In the new model proposed, the digestion and the adhesion to intestinal cell lines was simulated consecutively. This means that the trials described in sections 2.2–2.4 were carried out using the same bacterial culture of *Lbs. rhamnosus* GG ATCC 53103 and *A. muciniphila* ATCC BAA-835 from start to finish, without using fresh cultures in the different phases. For this purpose, the two strains were grown overnight at 37°C in 150 mL of MRS (Oxoid) and BHI broth (Oxoid), respectively. Bacterial suspensions of each strain were adjusted to the required concentration (about 10^8^ CFU/mL) with the McFarland turbidity following standard procedures, and counts were confirmed by 10-fold serial dilution and plating. The cultures were centrifuged at 8,000 rpm for 10 min at 4°C (Centrifuge 5415 R; Eppendorf, Hamburg, Germany), and the pellet, washed twice, was resuspended in the same volume of sterile saline solution. One mL of each strain was taken to perform microbial counts on the respective media at 37°C for 72 h under anaerobic conditions (AnaeroGen, Oxoid Ltd., Hampshire, United Kingdom).

### 2.2 Gastrointestinal transit simulation

The survival during simulated gastrointestinal transit (GIT) was evaluated following the protocol described by Vergalito et al. (2020). 33 mL of a sterile electrolyte solution (NaCl 6.2 g/L, KCl 2.2 g/L, CaCl_2_ 0.22 g/L, and NaHCO_3_ 1.2 g/L) simulating saliva and lysozyme from chicken egg white (Sigma-Aldrich) were added to sterile saline solution containing each bacterial strain, prepared as described above, at a final concentration of 0.01%. After a 2 min incubation, microbial counts were performed. The solution containing each bacterium was then divided into four sterile bottles (portions of 45 mL each). To simulate the gastric environment, 8.25 mL of electrolyte solution containing 0.3% pepsin from porcine gastric mucosa (final concentration) (Sigma-Aldrich) was added to each bottle. Two batches (consisting of three bottles each) were obtained for each strain: the pH was lowered to 2.0 in the first batch (batch pH 2) and to 3.0 in the second batch (batch pH 3) by adding 1.0 N HCl. Aliquots of each batch were collected after 30 and 90 min and used for microbial counts.

To simulate intestinal stress, oxygen was replaced by nitrogen in each bottle to achieve an anaerobic atmosphere, and the pH value was adjusted to 5.0 with a saturated sodium bicarbonate solution (8 g sodium bicarbonate in 100 mL distilled water, sterilised at 121°C for 15 min). Then 8.25 mL of a sterile electrolyte solution containing 0.45% porcine bile bovine and 0.1% pancreatin from porcine pancreas (final concentration, both produced by Sigma-Aldrich) were added to each bottle. The pH was then adjusted to 6.3 and slowly increased to 7.5 until the end of the intestinal stress test (4 h). Aliquots from each batch were collected and used for microbial counts. During the simulation of transit in the intestinal tract, the bottles were shaken at 150 rpm with digital orbital shaker (Heathrow Scientific) (Cueva et al., 2019). Solutions were freshly prepared each day and the entire study was conducted at 37°C.

At the same time as sampling for plate counts, aliquots (1.5 mL) were taken to perform the cell viability assays Live/Dead, described below (see section 2.5).

At the end of the GIT simulation, 1 mL of the bacterial suspension of both *Lbs. rhamnosus* GG and *A. muciniphila* was collected for microbial counts and 2 mL for the intestinal cell adhesion assay.

### 2.3 Propagation and maintenance of cell lines

Three immortalised human cell lines were used in this study: CaCo2 (ATCC HTB-37, human colon adenocarcinoma), HT-29 (ATCC HTB-38, human colon adenocarcinoma), both purchased from the American Type Culture Collection (ATCC, Rockville, MD, United States) and mucus secreting HT-29-MTX, purchased from the European Collection of Authenticated (ECACC) cell cultures (Health Security Agency, United Kingdom). Cells were cultured in 5% CO_2_ enriched atmosphere for CaCo2 and HT-29 cell lines and 8% for HT-29-MTX cell lines at 37°C in Dulbecco’s modified Eagle’s medium (DMEM; HyClone Laboratories Inc., Logan, UT, United States) supplemented with 10% (v/v) heat-inactivated foetal bovine serum (FBS; HyClone) (56°C for 30 min), 2 mM L-glutamine (Sigma-Aldrich, St. Louis, MO, United States), penicillin (100 U/mL) and streptomycin (100 mg/mL) (Biological Industries, Kibbutz Beth Haemek, Israel) in a 25 cm^2^ culture flask. The growth medium was replaced every other day until confluence was reached. Cells were harvested by adding 3 mL of 0.25% trypsin–EDTA solution at 37°C. After approximately 60% cell detachment, assessed using an inverted microscope (Olympus, model IMT2), 7 mL of complete DMEM was added to each flask. The cell suspensions were centrifuged at 1,200 rpm for 5 min at room temperature and the pellets were resuspended in complete DMEM (Duary et al., 2011). Instructions given by the Providers (ATCC and ECACC) were followed to obtain mature cells (15 days after seeding for CaCo2 cells and 21 days for both HT-29 and HT-29-MTX cells). Before each assay, the cells were re-observed under an inverted microscope to correctly assess confluency. Cell cultures of each line were divided into aliquots in six-well plates (concentration of 1 × 10^5^ cells/2 mL DMEM) and incubated as described above. When the cells reached confluence, the medium was changed daily until the adhesion assay. The depleted DMEM was removed 24 h before the adhesion assay and DMEM without antibiotics was added (Duary et al., 2011).

### 2.4 Adhesion assay and cell viability test

Strains of *Lbs. rhamnosus* GG ATCC 53103 and *A. muciniphila* ATCC BAA-835 were subjected to the adhesion assay immediately after undergoing GIT. For this purpose, aliquots of 2 mL for each strain, withdrawn at the end of the GIT simulation, were centrifuged (8,000 rpm for 15 min at 4°C), washed twice and resuspended in DMEM without antibiotics.

Each cell line was washed twice with 3 mL PBS (pH 7.4) and bacterial strains were added to six-well plates, incubated at 37°C in 5–8% CO_2_ (depending on the cell line) for 2 h. At the end of the incubation period, the DMEM was aspirated, and the monolayers were washed three times with sterile PBS. The cells in each well were lysed with trypsin–EDTA (0.25%) at 37°C.

To enumerate adherent bacteria, cell lysates were serially diluted with sterile saline solution (NaCl 0.9%) and plated on appropriate culture media. The percentage of adherence was calculated as follows (Valeriano et al., 2014):


$$\%\mathrm{Relative adhesion}=\left[\begin{array}{l} \left(CFU/\mathrm{mL}\;\mathrm{after adhesion}\right)/\\ \left(CFU/\mathrm{mL}\;\mathrm{before adhesion}\right) \end{array}\right]\;x\;100$$

For comparative purposes, cell adhesion assay was also performed on *Lbs. rhamnosus* GG ATCC 53103 and *A. muciniphila* ATCC BAA-835 not subjected to GI transit. In this case, fresh cultures of each strain, prepared as described in section 2.1, were centrifuged (8,000 rpm for 15 min at 4°C), washed twice and resuspended in sterile phosphate-buffered saline (PBS, pH 7) at 0.5 MacFarland Optical Density Scale (OD580, Multilabel Counter-PerkinElmer 1420, San Jose, CA, United States) to standardise the bacterial density to 10^8^ CFU/mL. Subsequent dilutions in DMEM without antibiotics resulted in final bacterial loads of 10^7^, 10^6^, 10^5^, 10^4^, 10^3^ and 10^2^ CFU/mL, confirmed by plate counting. Each bacterial suspension was then added to six-well plates and finally enumerated following the same protocol just described for strains subjected to GI transit. Additionally, cytotoxicity of all bacterial concentrations of *A. muciniphila* and *Lbs. rhamnosus* GG on the different cell lines was conducted using neutral red uptake assay. Briefly, cell lines, grown in 96 well plates in DMEM and allowed for overnight attachment, were then treated with different bacterial concentrations (from 10^8^ to 10^2^ CFU/mL) of *A. muciniphila* and *Lbs. rhamnosus* GG for 24 h. Cells were subsequently processed for neutral red assays for cytotoxicity measurement (Borenfreund and Puerner, 1985). The viability percentage was calculated as the absorbance at 540 nm of the treated cells (added bacteria) divided by the absorbance of the control cells [non-added bacteria (AB/AC × 100)].

All the assays were performed at least in triplicate. The adhesion percentage achieved by strains after simulated GIT was compared with that of strains which did not undergo the GI transit.

### 2.5 Live/dead assay

In addition to plate count, bacterial viability during GIT was assessed with the Live/Dead assay (BacLight™ Bacterial Viability Assay, L-13152, Invitrogen™, Molecular Probes®, Eugene, Oregon, United States). Equal volumes of SYTO 9 and propidium iodide (PI) were mixed as working solution to obtain a final concentration of each equal to 6 μM SYTO 9 and 30 μM PI. 140 μL of the mixture was added to a 500 μL aliquot of the sample. After incubation in the dark at room temperature for 15 min, all samples were washed to remove unbound dye and were filtered through Nuclepore Black Polycarbonate Membrane filters (Sigma Aldrich, Steinheim, Germany) with 0.2 μm pore size and 25 mm diameter. Each filter, containing labelled bacteria, was placed on a slide with two drops of BacLight mounting oil and covered with a glass coverslip. The number of live and dead bacteria was estimated from a count of 5 or more randomly chosen microscopic fields (1,000X); at least 400 bacteria were counted for each sample. Sample counts were performed using the Optic Ivymen® System model 3002-F epifluorescence microscope (Comecta, Abrera, Spain) under blue excitation beams (450–490 nm) with a 100 W mercury light source (Hu et al., 2017). Images were captured with a digital camera (ZLD Industrial Digital Camera) and associated software (ZLD ToupView version 3.7). Image analyses were performed using Fiji Software (version 2.9.0). The percentage of live cells was calculated as follows:


$$\%\mathrm{live cells}=\left[\mathrm{live cells}/\left(\mathrm{live cells}+\mathrm{dead cells}\right)\right]\;x\;100$$

Percentages were then converted to Log CFU/mL.

### 2.6 Database search for protein sequences of *Akkermansia muciniphila* interacting with human mucins

To study the interactions of *A. muciniphila* with human mucins, the GenBank (NCBI) and Uniprot (v. 2020_1) databases were consulted and used to explore the genome and proteome of *A. muciniphila* and PSI-BLAST (EMBL-EBI) to identify proteins with sequences homologous to *A. muciniphila* proteins involved in mucin-binding. CytoScape software (v3.10.1) was used to visualise and analyse the molecular interaction networks elucidated from the data obtained in the previous steps.

### 2.7 Statistical analysis

Statistical analysis and data visualisation were carried out in R environment (R Core Team, 2022). Data were evaluated for normal distribution using Shapiro–Wilk test and expressed as means ± standard deviation (SD). Wilcoxon test, unpaired Student’s *t*-test, Kruskal-Wallis test with Dunn’s test or one-way ANOVA followed by *post-hoc* Turkey Honestly Significant Difference (HSD) test for multiple comparisons were used to evaluate differences in adhesion rate or survival among groups. Graphics were generated with ggplot2 (Wickham, 2016) package. *p* values ≤ 0.05 were considered statistically significant.

## 3 Results

### 3.1 Assessment of survival to GIT simulation with plate count and live/dead assay

Considering that data deriving from plate counts were acquired in numeric form (Colony Forming Unit/mL, CFU/mL) while those from the Live/Dead assay were acquired as percentages and then transformed into Base-10 Logarithm of Colony Forming Unit/mL (Log CFU/mL), results described in this section refer only to cell viability reported on a logarithmic scale (Log CFU/mL) to keep the data cleaner and easier to understand.

**Figure 1:**
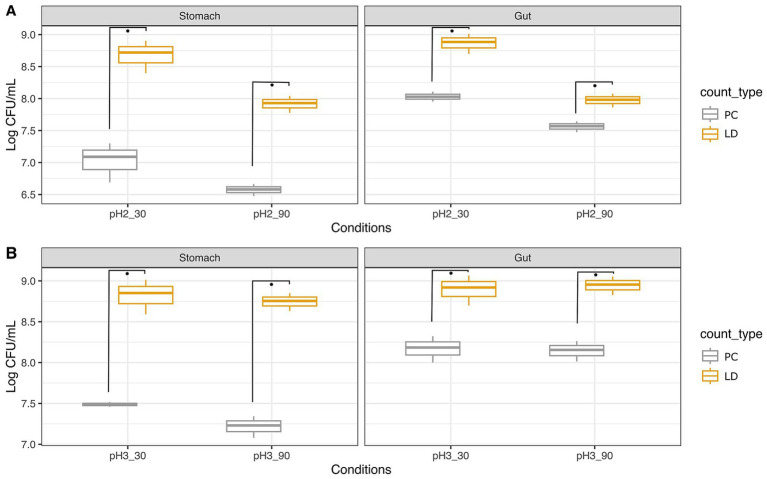
Survival of *Akkermansia muciniphila* ATCC BAA-835 to simulated GIT as ascertained with plate count (PC) and Live/Dead test (LD). Trials were performed at pH 2 **(A)** and pH 3 **(B)** for 30 and 90 min. Wilcoxon test was used to evaluate significant differences (*p* value ≤0.05) between groups. Asterisks indicate significant differences between groups calculated on three replicates.

**Figure 2:**
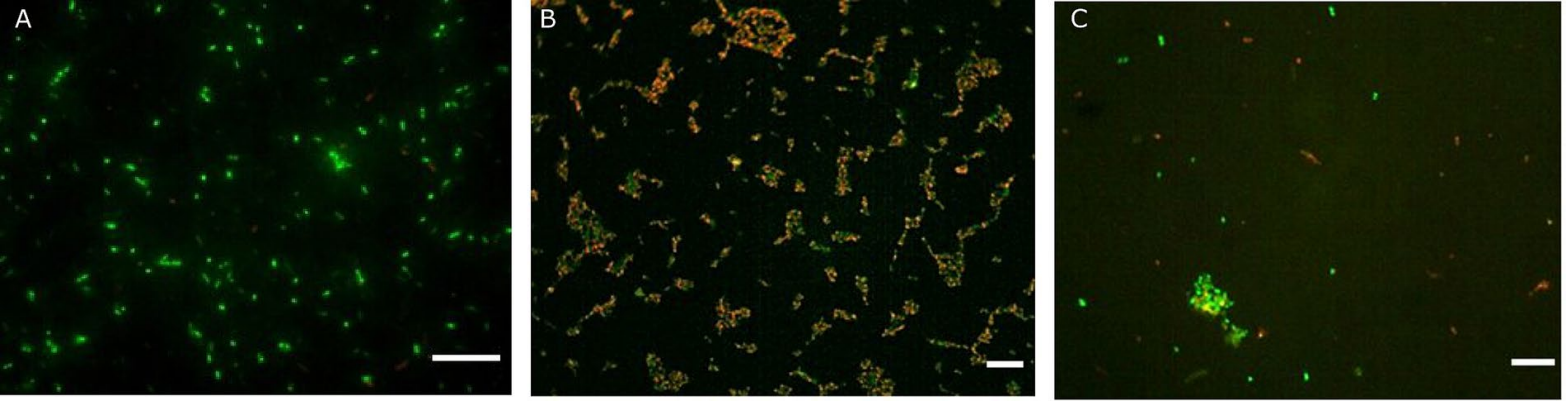
Fluorescence microscopy representative images of *Akkermansia muciniphila* ATCC BAA-835 obtained with the Live/Dead assay prior simulated GIT **(A)**, after gastric conditions at pH 2 for 30 min **(B)** and at the end of the intestinal transit **(C)**. Scale bar: 10 μm.

The study compared the survival rates of *A. muciniphila* ATCC BAA-835 and *Lbs. rhamnosus* GG ATCC 53103 through simulated GIT using both plate count and fluorescent probes. In general, *A. muciniphila* showed much higher survival rates than *Lbs. rhamnosus* GG. Moreover, fluorescent probes (Live/Dead, LD) always returned higher percentages of microbial charges compared to plate counts (PC). Figure 1 shows the results of the simulated GIT of *A. muciniphila* ATCC BAA-835, while Figure 2 illustrates some images captured with fluorescence microscopy during the trial.

The initial load of *A. muciniphila* was about 8.95 Log CFU/mL (Supplementary Figure S1), decreasing to 7.09 Log CFU/mL after 30 min in gastric conditions at pH 2 (Figure 1A). The LD assay performed in this step indicated counts of 8.72 Log CFU/mL, higher than those detected with PC. The transition to intestinal conditions showed an increase to 8.03 Log CFU/mL and 8.88 Log CFU/mL, as determined by the PC and the LD assays, respectively. The same test was repeated at pH 2, but with a longer permanence time in gastric conditions (90 min instead of 30 min). In this case, counts of 6.58 Log CFU/mL and 7.93 Log CFU/mL were registered with PC and LD, respectively. After the intestinal transit, counts increased to 7.57 Log CFU/mL and approximately 8 Log CFU/mL as ascertained with the two methods.

As expected, the same experiment repeated at pH 3 gave better results in terms of bacterial survival. Specifically, after 30 min at pH 3 (Figure 1B), counts of *A. muciniphila* were 7.49 Log CFU/mL with PC and 8.85 Log CFU/mL with the LD assay. In intestinal conditions, loads were 8.19 (PC) and 8.92 (LD) Log CFU/mL. By applying a longer residence time, that is, 90 min at pH 3, *A. muciniphila* exhibited loads of 7.23 Log CFU/mL and 8.75 Log CFU/mL by PC and LD assay, respectively. Counts increased to 8.16 (PC) and 8.95 (LD) Log CFU/mL in intestinal conditions.

**Figure 3:**
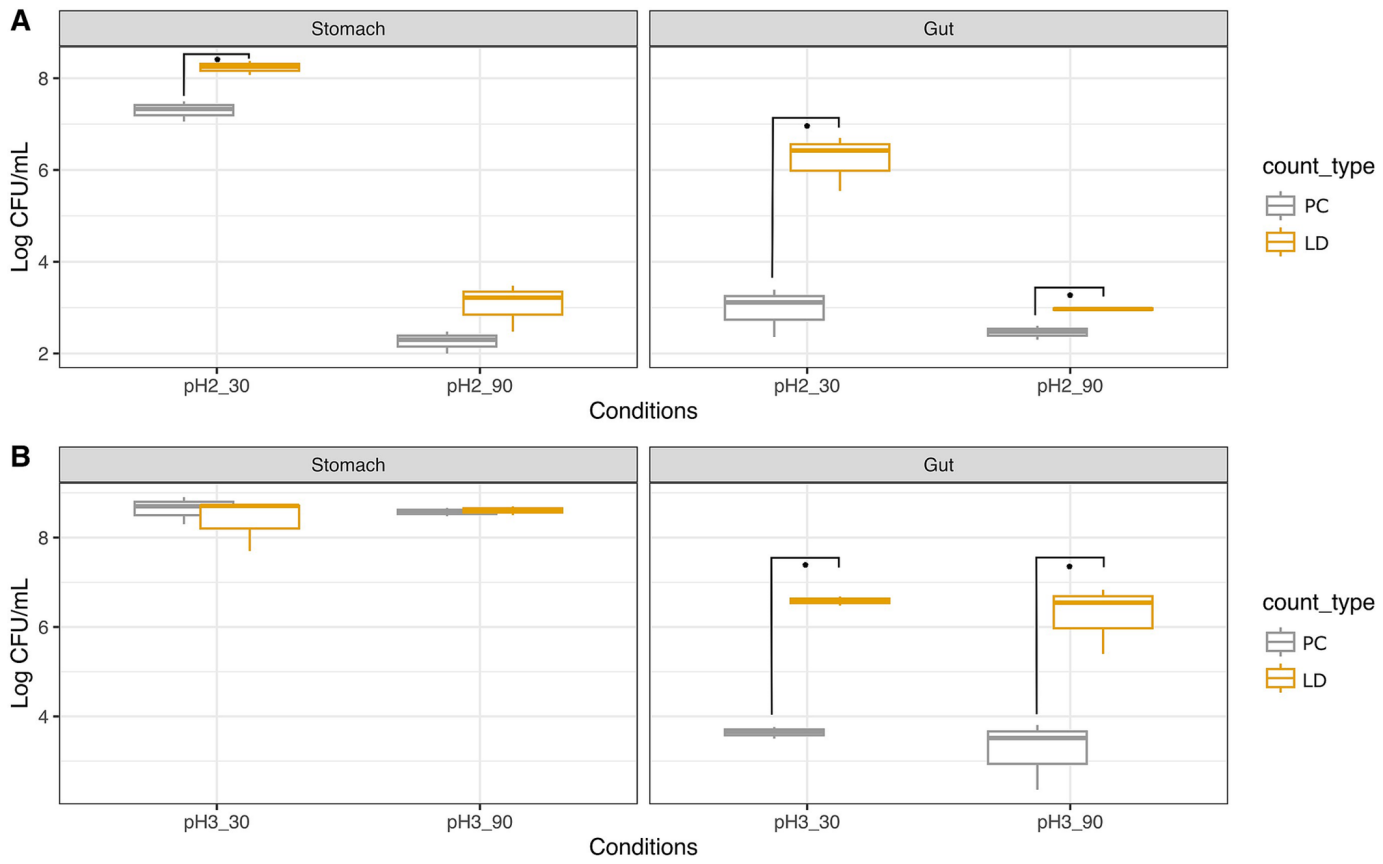
Survival of *Lacticaseibacillus rhamnosus* GG ATCC 53103 to simulated GIT as ascertained with plate count (PC) and Live/Dead test (LD). Trials were performed at pH 2 **(A)** and pH 3 **(B)** for 30 and 90 min. Wilcoxon test was used to evaluate significant differences (*p* value ≤0.05) between groups. Asterisks indicate significant differences between groups calculated on three replicates.

**Figure 4:**
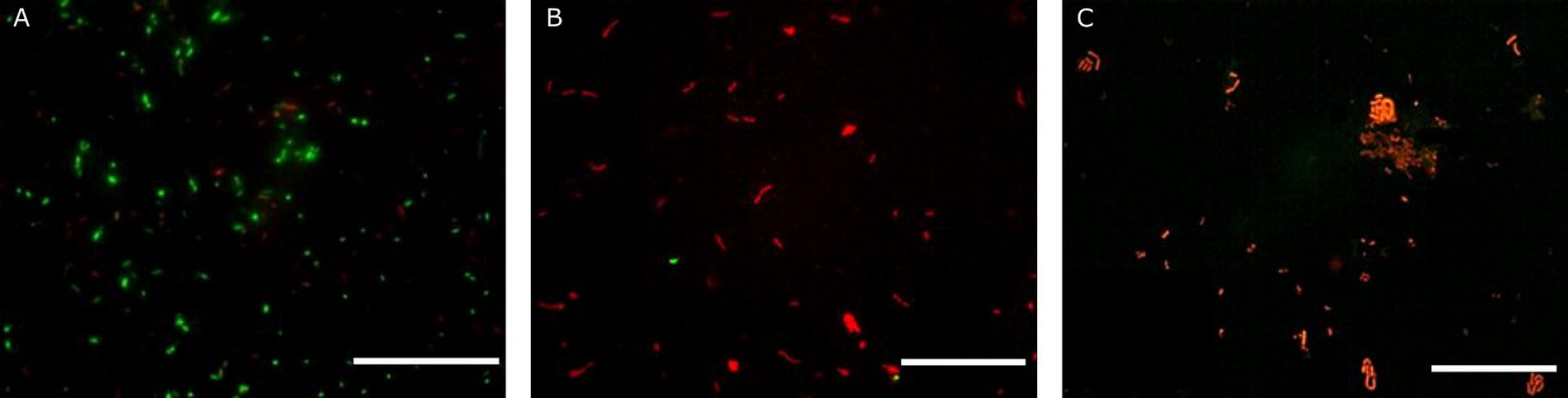
Fluorescence microscopy representative images of *Lacticaseibacillus rhamnosus* GG ATCC 53103 obtained with the Live/Dead assay prior simulated GIT **(A)**, after gastric conditions at pH 2 for 30 min **(B)** and at the end of the intestinal transit **(C)**. Scale bar: 50 μm.

For comparative purposes, the experiment was repeated on the probiotic strain *Lbs. rhamnosus* GG ATCC 53103 (Figure 3). Figure 4 illustrates some images captured with fluorescence microscopy during the trial.

*Lacticaseibacillus rhamnosus* GG showed consistently higher loads assessed with the LD assay compared to PC. Specifically, the initial load was about 8.72 Log CFU/mL (Supplementary Figure S2), and after 30 min at pH 2, counts of 7.33 Log CFU/mL were recorded with PC, while the LD assay indicated 8.25 Log CFU/mL. After 90 min at pH 2, counts decreased significantly to 2.30 Log CFU/mL and 3.22 Log CFU/mL, as detected by PC and LD assay, respectively. In intestinal conditions, counts further reduced to 3.11 and 2.48 Log CFU/mL with PC, while the LD assay showed higher loads of 6.43 and 2.97 Log CFU/mL for pH2 30 min and 90 min, respectively.

As pointed out earlier for *A. muciniphila*, also in the case of *Lbs. rhamnosus* GG the survival rate resulted higher at pH 3 than pH 2. In fact, after 30 min at pH 3, charges of about 8.7 Log CFU/mL were registered with both PC and LD assay. After 90 min, 8.58 Log CFU/mL were detected with PC, slightly lower than those given by the LD (8.61 Log CFU/mL). Simulation of intestinal conditions after stomach permanence at pH 3 showed comparable counts of 3.65 Log CFU/mL and 3.52 Log CFU/mL after 30 and 90 min, respectively, when PC was adopted, while higher loads of 6.59 Log CFU/mL and 6.54 Log CFU/mL, respectively, were noted with the LD assay.

### 3.2 Cytotoxicity and adhesion to human intestinal cell lines

**Figure 5:**
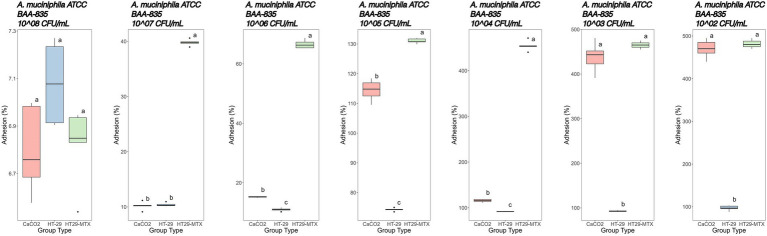
Adhesion percentages of *Akkermansia muciniphila* ATCC BAA-835 added at different concentrations (from left to right: 8–2 Log CFU/mL) to CaCo2, HT-29, and HT29-MTX cell lines. Data with different superscript letters are significantly different (*p* value ≤0.05) according to results of Tukey’s *post hoc* tests. Boxplots visualize five summary statistics of five replicates: the median, two hinges and two whiskers. Lower and upper hinges correspond to the first and third quartiles. Upper whisker extends from the hinge to the largest value no further than 1.5 × IQR from the hinge (where IQR is the distance between the first and third quartiles). Lower whisker extends from the hinge to the smallest value at most 1.5 × IQR of the hinge. Data beyond the end of the whiskers are ‘outlying’ points and are plotted individually.

**Figure 6:**
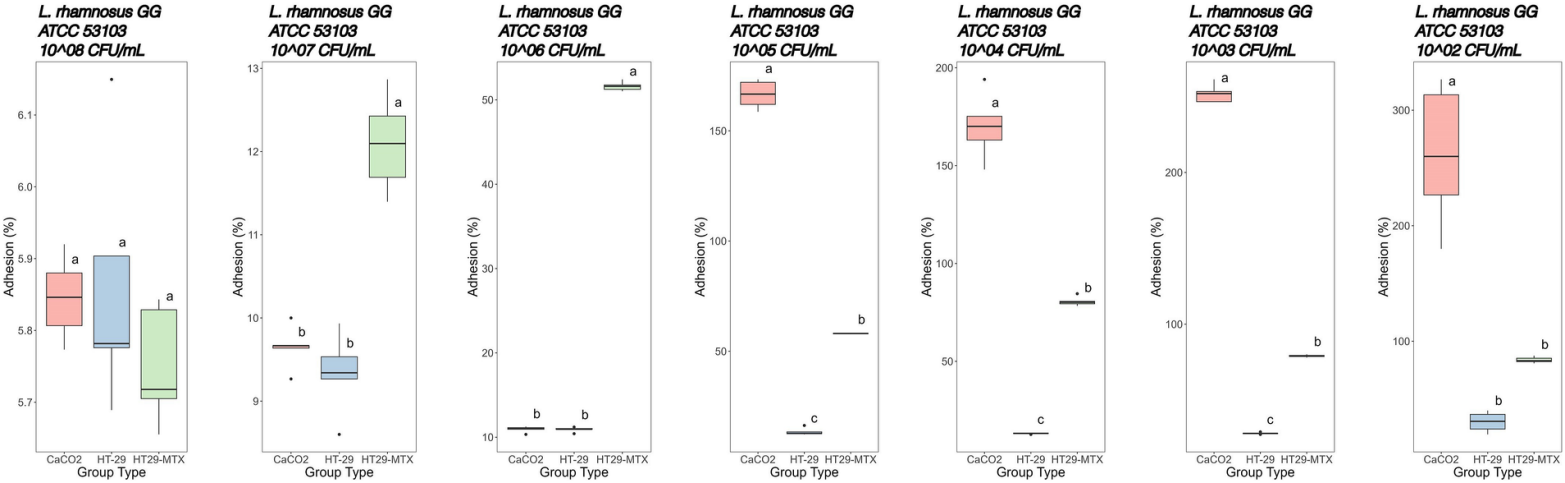
Adhesion percentages of *Lacticaseibacillus rhamnosus* GG ATCC 53103 added at different concentrations (from left to right: 8–2 Log CFU/mL) to CaCo2, HT-29 and HT29-MTX cell lines. Data with different superscript letters are significantly different (*p* value ≤0.05) according to results of Tukey’s *post hoc* tests. Boxplots visualize five summary statistics of five replicates: the median, two hinges and two whiskers. Lower and upper hinges correspond to the first and third quartiles. Upper whisker extends from the hinge to the largest value no further than 1.5 × IQR from the hinge (where IQR is the distance between the first and third quartiles). Lower whisker extends from the hinge to the smallest value at most 1.5 × IQR of the hinge. Data beyond the end of the whiskers are “outlying” points and are plotted individually.

Immediately after the simulated GIT, the bacteria were subjected to adhesion tests on different cell lines. Moreover, in order to compare the results with the control, it was necessary to conduct the adhesion assay on the same bacterial strains not subjected to GIT. The adhesion screening was preceded by the cytotoxicity assay performed on all cell lines by using different bacterial concentrations, from 8 to 2 Log CFU/mL. No bacterial concentrations showed cytotoxicity (Supplementary Figure S3). With regard to adhesion tests (Figures 5, 6), we noted that the adhesion rate of *A. muciniphila* and *Lbs. rhamnosus* GG to CaCo2, HT-29 and HT-29-MTX cells was inversely proportional to the added bacterial concentration. Additionally, the highest bacterial concentration used for each tested strain, that is, 8 Log CFU/mL, did not display significant differences (*p* value >0.05) in the adhesion percentage to the three cell lines (Figures 5, 6). The adhesion of *A. muciniphila* to HT-29 and HT-29-MTX cell lines was always significantly higher than that presented by *Lbs. rhamnosus* GG (*p* value ≤0.05), except for the combination 6 Log CFU/mL/HT-29 cell line, where the adhesion rate was not significantly different between tested strains (see also Supplementary Table S1).

More specifically, adhesion of *A. muciniphila* to the CaCo2 cell line showed percentages of 6.80, 10.20 and 15.20% at initial concentrations of 8, 7 and 6 Log CFU/mL, respectively (Figure 5). Starting with concentrations of 5 Log CFU/mL, adhesion percentages higher than 100% were recorded, and the highest adhesion was detected when 2 Log CFU/mL were added (470%).

A similar trend was observed with the cell line HT-29 (Figure 5). However, the adhesion was generally lower in comparison with CaCo2 cells, especially when microbial loads below 6 Log CFU/mL were used. In fact, at initial concentrations of 8, 7 and 6 Log CFU/mL, an adhesion of 7.08, 10.40 and 11.00%, respectively, was detected. Using 5, 4, 3 and 2 Log CFU/mL, increasing adhesion rates were recorded (about 74, 92 93 and 97%, respectively).

The adhesion of *A. muciniphila* to the HT-29-MTX cell line showed the highest adhesion rate starting from 7 Log CFU/mL added cells. In fact, as previously stated, the initial concentration of 8 Log CFU/mL was substantially similar, in terms of adhesion percentage, to that reported with the other tested cell lines (6.82%). However, starting from 7 Log CFU/mL bacterial concentration up to 3 Log CFU/mL, we found significantly higher adhesion percentages in comparison with those detected by using the other cell lines (Figure 5).

The adhesion of *Lbs. rhamnosus* GG ATCC 53103 to CaCo2 cells (Figure 6) showed percentages of 5.84, 9.65 and 10.90% at initial concentrations of 8, 7 and 6 Log CFU/mL, respectively. Hereinafter, adhesion percentages higher than 100% were recorded.

The HT-29 cell line in contact with *Lbs. rhamnosus* GG showed adhesion rates of 5.86, 9.34 and 10.91% when 8, 7 and 6 Log CFU/mL bacterial concentrations, respectively, were used. Lower concentrations in the range 5–2 Log CFU/mL showed adhesion percentages increasing from 13.50 to 30.10% (Figure 6; Supplementary Table S1).

Finally, the adhesion of *Lbs. rhamnosus* GG to HT-29-MTX cells was lower (*p* value ≤0.05) than that detected with CaCo2 cells, except when 7 Log CFU/mL bacterial concentration was used and, more markedly, with 6 Log CFU/mL (Figure 6; Supplementary Table S1).

### 3.3 Comparison of the adhesion to human intestinal cell lines of strains subjected and non-subjected to simulated GIT

**Figure 7:**
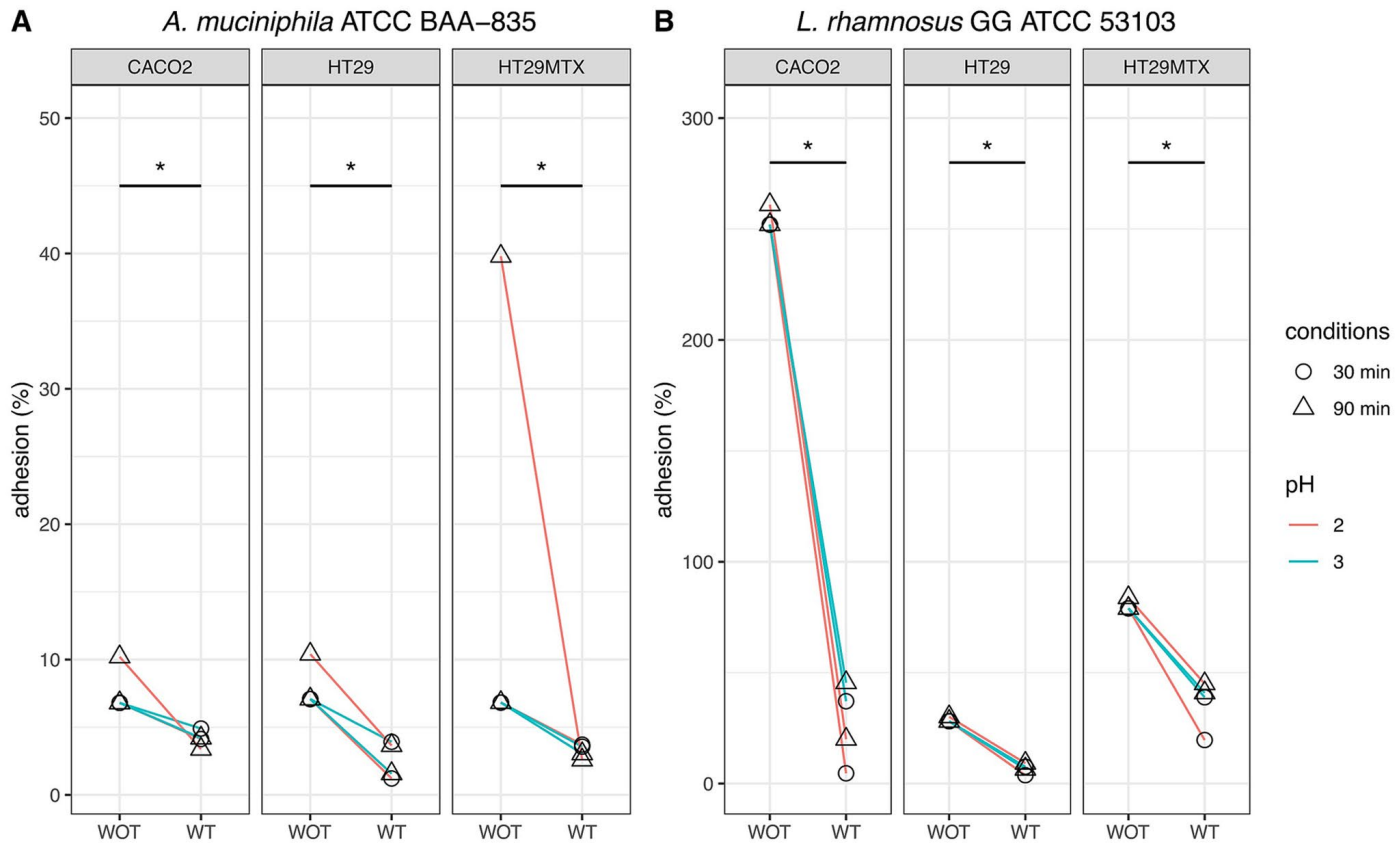
Comparative analysis of adhesion to human intestinal CaCo2, HT-29, and HT-29-MTX cell lines of *Akkermansia muciniphila* ATCC BAA-835 **(A)** and *Lacticaseibacillus rhamnosus* GG ATCC 53103 **(B)** subjected (WT) or not (WOT) to simulated GIT. Wilcoxon test was used to evaluate significant differences (*p* value ≤0.05) between groups. Asterisks indicate significant differences between groups.

Comparative analysis of adhesion assays performed on *A. muciniphila* and *Lbs. rhamnosus* GG undergoing or not simulated GIT revealed notable differences (Figure 7). In general, a consistent reduction of adhesion was observed for bacteria subjected to simulated GIT stress. This reduction was particularly evident in some conditions. For instance, *A. muciniphila* previously subjected to simulated GIT with a gastric phase at pH 2 for 90 min, showed an adhesion rate of 2.57% to HT-29-MTX, extremely low if compared to the 39.8% adhesion detected for the same bacterium non-subjected to simulated GIT. For all the other conditions, lower differences (between 1.9 and 6.8%) were detected in the adhesion of *A. muciniphila*, subjected and non-subjected to simulated GIT, to the different cell lines.

Similarly, *Lbs. rhamnosus* GG previously subjected to simulated GIT with a gastric phase at pH 2 for 30 and 90 min, showed adhesion rates of 4.62 and 20% to CaCo2 cells, respectively, whereas more than 250% adhesion was detected when the strain was tested without simulated GIT. For the same strain evaluated after simulated GIT under gastric phase at pH 3 for 30 and 90 min, an adhesion to CaCo2 cells of 37.1 and 45.6% was registered, respectively, whereas more than 250% adhesion was detected in the tests without simulated GIT.

As for the other conditions, adhesions evaluated after simulated GIT were always significantly lower than those detected for the same strain non-subjected to GIT. The highest differences, apart from those just described, were highlighted for *Lbs. rhamnosus* GG subjected to simulated GIT under all gastric conditions (pH 2 or pH 3, for 30 or 90 min): the adhesion rates to HT-29 and to HT-29-MTX ranged from-20.5% to-24.3%, and from-38.1% to-59.4%, respectively, compared to the control (strain non-subjected to simulated GIT). These findings suggest a significant influence of GI transit conditions on the adhesion of *Lbs. rhamnosus* GG to human intestinal cell lines.

### 3.4 Protein interactions of *Akkermansia muciniphila* with human mucins

To further explore the ability of *A. muciniphila* to persist in the host gut, a bioinformatic study was performed by exploring the genome and proteome of the strain ATCC BAA-835/ DSM 22959/JCM 33894/BCRC 81048/CCUG 64013/CIP 107961/Muc, with a particular focus on mucins. The genome consists of a circular chromosome of 2,664,102 bp with an average GC content of 55.8%, a total of 2,210 genes and 12 pseudogenes. *A muciniphila* also possesses two CRISPR (clustered regularly interspaced short palindromic repeats) loci that represent primitive and adaptive primitive immune systems in bacteria against invading agents such as bacteriophages or plasmids. Two genes associated with resistance to *β*-lactam antibiotics (Amuc_0106 and Amuc_0183), a gene coding for an antibiotic resistance protein 5-nitroimidazole (Amuc_1953) and a gene coding for a putative antibiotic biosynthesis monooxygenase (Amuc_1805). The genome of this bacterium contains more than 300 genes (about 11%) for supposed proteins involved in mucin degradation, but the nature and function of most of these proteins are unknown. The proteome of *A. muciniphila* (strain ATCC BAA-835/DSM 22959/JCM 33894/BCRC 81048/CCUG 64013/CIP 107961/Muc) includes 2,137 proteins. Proteomic analysis revealed over 80 GI mucin-degrading enzymes, such as glycosidases, sulfatases, proteases and sialidases, hexosaminidases, galactosidase, known as ‘mucinases’ (Supplementary Table S2).

The analysis also detected the presence of a mucin-associated surface protein (MASP; UniProtID: B2UQG4, is encoded by the Amuc_0866 gene) and a pili assembly protein (PilO; UniProtID: B2UQG4, encoded by the Amuc_1100 gene). The sequence matches of the MASP and PilO proteins were searched in depth in the protein database. More than 500 similar protein sequences were found, mainly belonging to the *phylum* Verrucomicrobia and the genus *Akkermansia*. Similar proteins with very low identity percentages (<20%) were also found in bacteria of the *phylum* Firmicutes and Armatimonadota.

The GroEL protein of *A. muciniphila* has an identity percentage of 60.07% with the GroEL protein of *Lactobacillus johnsonii*. This suggests that this protein might have the same function in *A. muciniphila*, i.e., interacting with human mucins in the stomach, preventing *Helicobacter pylori* from binding with the mucins MUC5AC and MUC1 and promoting their aggregation and elimination (Vanden Brink et al., 2000; Bergonzelli et al., 2006; Lillehoj et al., 2012).

**Figure 8:**
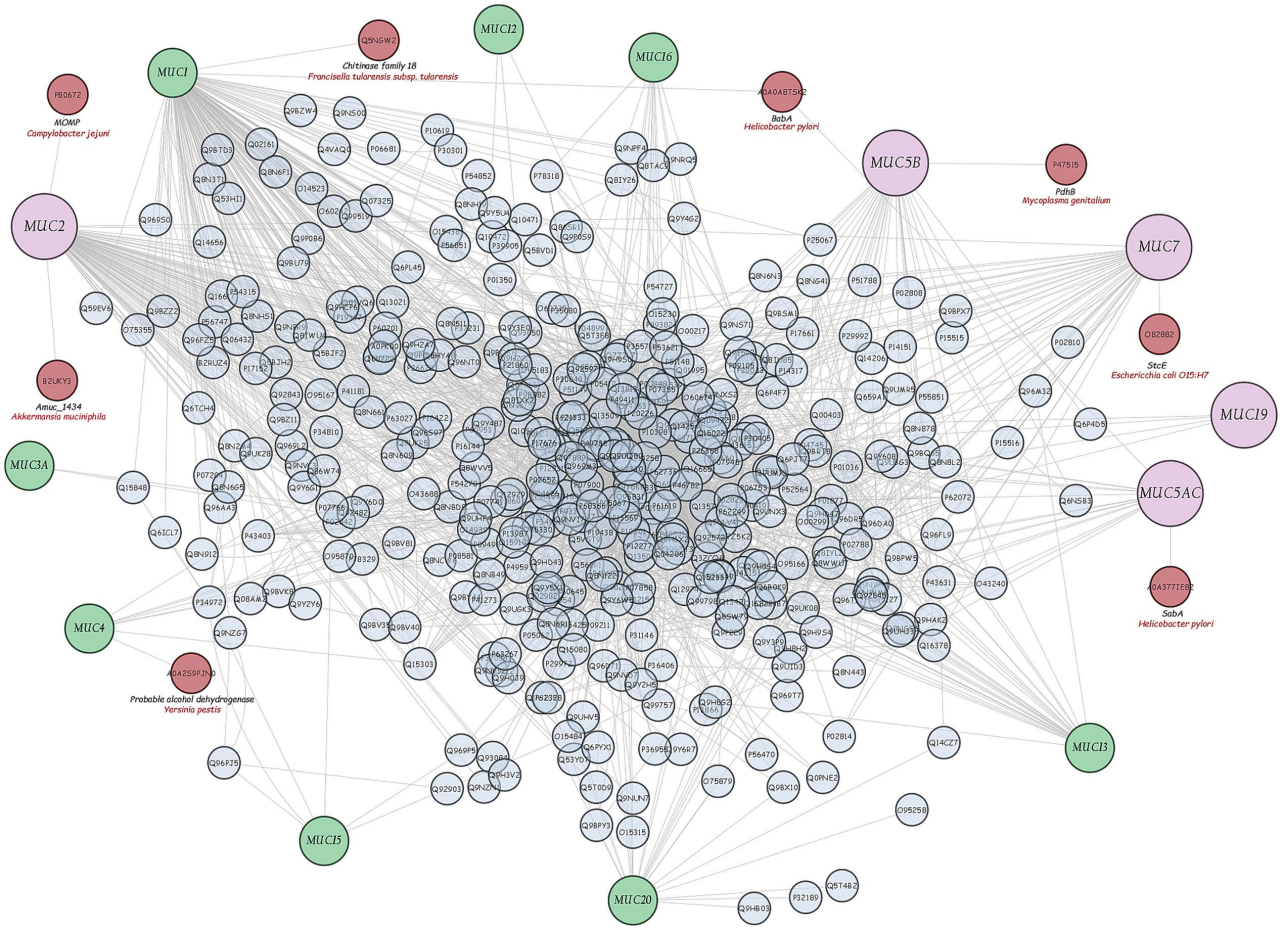
Graphical network of interactions between human mucins and human and bacterial proteins found in literature and online databases (Cytoscpae v3.10.1 software).

Following the integration of a database of interactions between human mucins and bacterial proteins [found in the literature and online databases—Pathogen-Host Interaction Search Tool (PHISTO), a graphical interaction network was created using the Cytoscape v3.10.1 software] (Figure 8). Only the Amuc_1434 DUF6268 domain-containing protein of *A. muciniphila* is able to promote adhesion to the LS174T (colon cancer) cell line, which expresses high levels of MUC2, demonstrating the ability of this bacterium to bind MUC2 in the mouse colon (Meng et al., 2020). Since human mucin-2 shares more than 76% identity with mouse mucin-2, it can be assumed that the Amuc_1434 protein of *A. muciniphila* also binds to the human MUC2 protein.

## 4 Discussion

The effectiveness of probiotic microorganisms in providing health benefits to the host depends on the number of viable bacteria that successfully pass through the GI tract and reach the colon (Latif et al., 2023). In our study, fluorescent probes always returned higher charges than plate counts. Some researchers have suggested that a portion of the bacterial population may enter a state known as viable but non-culturable when subjected to gastric stress (Possemiers et al., 2010). Within the gut environment, under less hostile conditions, some VBNC cells could revert to a culturable state, potentially accounting for the observed increase in viability following simulation of intestinal transit (de Palencia et al., 2008; Faye et al., 2012). Additionally, de Palencia et al. (2008) noted that gastric stress induced by low pH levels leads to the formation of cell aggregates, which may result in the formation of a single colony during viable plate counts, thus leading to an underestimation of viable cells. Conversely, these aggregations tend to dissipate as pH conditions become more conducive, such as those encountered during intestinal transit. Similar behaviour during simulated transit has already been observed by other authors for LAB (Possemiers et al., 2010; Faye et al., 2012; Succi et al., 2017). The images reported in Figure 2 clearly display the behaviour of *A. muciniphila*. Indeed, cell aggregates in the gastric phase (Figure 2B) show red-stained outer bacterial cells protecting the green inner cells. This protective mechanism allowed most of the *A. muciniphila* cells, sheltered within the aggregates, to arrive viable in the gut (Figure 2C), where the lower environmental pressures permitted the cells to recover. This protective mechanism is lacking in *Lbs. rhamnosus* GG. In fact, no aggregates are present in Figure 4B and this bacterial strain was unable to efficiently cross the gastric barrier.

After this phase, *A. muciniphila* was further studied for its ability to adhere to the intestinal epithelium using tissue cultures, which represent the most widespread and versatile models for this type of test (Bermudez-Brito et al., 2013; Martínez-Maqueda et al., 2015). Enterocytes and globet cells constitute the two major cell types of the intestine, and CaCo2 and HT-29 cell lines, both deriving from colon adenocarcinoma, display similar structural and functional features of enterocytes. Specifically, HT-29 cells are characterised by a high degree of heterogeneity due to the formation of absorptive and goblet cells mixture. In contrast to HT-29, CaCo2 cells exhibit many properties of the small intestinal epithelium when grown in culture. They form a well-differentiated polarised monolayer of columnar absorptive cells that express brush border with typical small intestinal enzymes and transporters. However, this cell line lacks the typical mucus layer of the intestinal epithelium. The cell line HT-29-MTX derives from HT-29 cells. They differentiate into mucus secreting globet cell types during cultivation, forming a mucus layer on the top of the epithelial cells resembling the colon. In our research, we used all the three highly characterised intestinal models, i.e., the CaCo2, HT-29 and HT-29-MTX cell lines (Haddad et al., 2023), to test the adhesive capacity of *A. muciniphila*, as this characteristic is still considered one of the main selection criteria for probiotics (FAO/WHO, 2020). In a previous study by Reunanen et al. (2015), the adhesion of *A. muciniphila* and *Lbs. rhamnosus* GG was assessed on CaCo2 and HT-29 cells. In line with our results, they reported a greater adhesion to CaCo2 cells than that detected to the HT-29 cell line for both *A. muciniphila* and *Lbs. rhamnosus* GG. In the same work, Authors reported the ability of *A. muciniphila* to bind both undifferentiated and mature enterocytes, strengthening their integrity. To the best of our knowledge, no information is available on the adhesion ability of *A. muciniphila* to a mucus secreting cell line, that is, HT-29-MTX. In our study, *A. muciniphila* showed, in most cases, better adhesion to all used cell lines than *Lbs. rhamnosus* GG, and the adhesion of *A. muciniphila* to HT-29-MTX cells was always higher than that detected by using HT-29 and CaCo2 cells. This result could be due to the ability of this bacterium to bind mucus (Karamzin et al., 2021) and to use mucin as the sole carbon and nitrogen source (van Passel et al., 2011), even if these aspects are still debated. In fact, the high adhesion rate detected for *A. muciniphila* to non-and low-mucus producing CaCo2 and HT-29 cell models, in particular to CaCo2 cells, still demonstrated that the attachment is not necessarily mediated by the presence of a mucus layer. In order to shed further light on this aspect, in this study we used bioinformatics approach by exploring the genome and proteome of strain ATCC BAA-835 and, in particular, the interactions between bacterial proteins and human mucins, as the ability of intestinal bacteria to physically interact with mucin is still considered as related to their ability to utilise it (Nishiyama et al., 2024). The analysis of *A. muciniphila* proteome revealed the lack of canonical mucin-binding domains involved in the adhesion to the intestinal mucus layer, whereas about 80 enzymes for mucin degradation were found. With regard to the Amuc_1434 encoded by *A. muciniphila*, in our bioinformatic study it was the sole protein correlated with human MUC2 degradation (Figure 8). This protein is currently the subject of several studies, as it may play an important role in colorectal cancer treatment (Wang and Tang, 2024). However, the functional properties of putative mucinolytic proteins and their role in mucin binding or degradation are still unclear (Meng et al., 2020). Similar results were obtained by other Authors. For instance, in past years, van Passel et al. (2011) sequenced and annotated *A. muciniphila* genome, showing the presence of numerous candidate mucinase-encoding genes, but not of genes encoding canonical mucus-binding domains. The study of Png et al. (2010) showed the good ability of *A. muciniphila* to degrade both porcine mucin and human MUC2 after 48 h of culture. Derrien et al. (2010) highlighted the presence of numerous mucin-degrading enzymes identified in the human digestive tract related to *A. muciniphila*, including *α*-and *β*-D-galactosidase, α-L fucosidase, α-and β-N-acetylgalactosaminidase, β-Nacetylglucosaminidase, neuraminidase and sulfatase, but no information regarding the mechanisms of its mucus binding was reported. In a recent work by Zhang et al. (2019), a critical analysis of the current knowledge on *A. muciniphila* was given, but no information on its adhesion ability was furnished. Only a few studies investigated the role of some *A. muciniphila* ATCC BAA-835 proteins as mucus-binding candidates in mice. In the study by Yoon et al. (2021), the mucinase Amuc_1631 protein was shown to bind intercellular adhesion molecule 2 (ICAM2) and induced an increase in thermogenesis and secretion of glucagon-like peptide-1 (GLP-1), a therapeutic target for type 2 diabetes. These results suggest that knowledge about the role of mucin in the adhesion process of *A. muciniphila* is still limited and further research is needed.

On the other hand, by using three different cell lines, our study showed the ability of this bacterium to adhere either to the mucin covering the epithelial cell layer or directly to the enterocytes, explaining its detection in all parts of the intestinal tract (Derrien et al., 2010). Moreover, cell viability tests, conducted on the three cell lines treated with different concentrations of *A. muciniphila* and *Lbs. rhamnosus* GG, confirmed the absence of cytotoxicity of the two bacteria even when used at the highest concentration (Reunanen et al., 2015; Steele, 2022). The use of different bacterial concentrations provided a further information on the adhesion to intestinal cell lines in terms of binding affinity to cells. Our results highlighted low adhesion rates when bacterial concentrations were between 8 and 7 Log CFU/mL, with scarce differences between cell lines as well as between tested bacteria. Important differences were, however, appreciated starting from concentrations of 6 Log CFU/mL. This fact seems to confirm that in this kind of studies it is important to not saturate the cells binding sites, otherwise a progressively smaller fraction of bacterial cells will bind, and a lower adhesion rate will be observed (Ouwehand and Salminen, 2003). However, the adhesion test carried out in our study was performed when the monolayer of cells reached the confluence stage. Under these conditions, each bacterium could bind to tested cells. One possible explanation for the inverse correlation between bacterial concentration and adhesion was given by Lee et al. (2000), who hypothesised that multiple adhesion sites could be involved depending on the number of bacteria: when the bacterial concentration is low, adhesion to a maximum number of sites is possible; when bacterial concentration is high, a minimum number of adhesion sites are involved. On the other hand, Dimitrov et al. (2014) pointed out that several lactic acid bacteria bind eukaryotic cells using one or more adhesive factors, and this observation could also be considered to explain the lower adhesion of the strains we tested when analysed at high concentrations. The results obtained in our study confirm that higher bacterial concentrations of *A. muciniphila* and *Lbs. rhamnosus* GG showed lower adhesion rates than those recorded for lower concentrations, as observed for all cell lines. This research also highlighted that adhesion was generally greater than 100% for bacterial concentrations between 5 and 2 Log CFU/mL. This was particularly evident for *A. muciniphila* assessed on CaCo2 and HT-29-MTX cells and for *Lbs. rhamnosus* GG tested on CaCo2 cells. This is probably due to an increase in bacterial charge after 2 h of incubation, that is, the time generally required to perform the adhesion test (Ouwehand and Salminen, 2003). Obviously, this behaviour is not expected in human intestinal conditions, where a harsh environment is present.

In our study, we noted that strains subjected to simulated GIT change their *in vitro* adhesion properties to human intestinal cell lines. Indeed, the adhesion after transit of *A. muciniphila* and *Lbs. rhamnosus* GG was significantly lower than that assessed without transit. Similar results were reported in the study by de los Reyes-Gavilán et al. (2011) who examined the adhesion capacity of *Bifidobacterium* strains after simulation of GIT using human gastric juices. The reduced adhesion of the studied strains to human cell lines after simulated GIT could be induced by the various environmental stresses to which they are subjected during the trial. Loss of viability has a direct impact on the ability of bacteria to adhere to human cell lines (de los Reyes-Gavilán et al., 2011). Moreover, the exposure to GIT conditions can alter the surface characteristics of bacteria, and changes in surface proteins or carbohydrate structures can have an impact on the interaction between bacteria and cell lines. Specifically, during simulated GIT, promising probiotics are primarily exposed to saliva rich in lysozyme, which however has little impact on the survival rates and adhesion properties (Han et al., 2021). The first environment really capable of negatively influencing the survival of probiotics (or putative probiotics) and, consequently, their performance, is the stomach, where acidic gastric fluids are lethal to most bacteria due to the influx of hydrogen ions (H^+^) causing reduction of the bacterial cytoplasmic pH and malfunction of ATPase proton pumps (Yao et al., 2020). Furthermore, pepsin has long been known for its negative effect on bacterial protein structures involved in the adhesion process (Tuomola et al., 2000). Some studies highlighted that *A. muciniphila* may activate many acid resistance systems implying all the relevant genes for GABA production, including Amuc_0372 (glutamate decarboxylase), Amuc_0037 (amino-acid permease), and Amuc_0038 (glutaminase) (Ottman, 2015; Konstanti et al., 2024).

Resistant bacteria that reach the small intestine will find conditions more favourable to their survival in terms of pH (around 6.0–7.0), but the enzymatic richness of the intestinal juices and the presence of bile salts can still have negative effects on the adhesion, especially by damaging the bacterial cell membrane and DNA (Yao et al., 2020). In several lactic acid bacteria and bifidobacteria, exopolysaccharides are secreted to the environment around the cells as a protection strategy against the effect of low pH, pancreatic enzymes and bile salts (Castro-Bravo et al., 2018). In *A. muciniphila*, this mechanism of protection is not present, but a putative bile acid transporter gene annotated in its genome (locus Amuc_0139) could potentially be involved in the export of bile acid outside the cell (Geerlings et al., 2018). Once in the colon, competition with commensal bacteria for nutrients and adhesion sites pose further challenges, and only the most resistant bacteria will be able to colonise the intestinal mucosa and exert positive effects on the host.

In addition to the resistance mechanisms just described for *A. muciniphila*, our study also highlighted the ability of this bacterium to form aggregates in the gastric environment to protect itself by acidic juices and pepsin. This trait was also studied by Becken et al. (2021), who noted the ability to self-aggregate in some *A. muciniphila* isolates, but not in the reference strain Muc^T^. Indeed, in our previous study (Cozzolino et al., 2020) we already highlighted a high auto-aggregation in *A. muciniphila* DSM 22959, but this character was only studied in relation to the adhesion ability of this strain. In the present study, to the best of our knowledge, for the first time we observed this feature in simulated GIT as a defence mechanism against gastric damages. In our aforementioned study, we also highlighted the poor ability of *A. muciniphila* to form biofilm, as has also been shown by other Authors (Lv et al., 2024), thus suggesting that biofilm formation is not adopted by this bacterium as a strategy to increase permanence and adhesion in the gut.

The mode of action of probiotic or potentially probiotic microorganisms to survive gastrointestinal stressors is therefore a critical requisite to ensure sufficient adhesion. Simulation of GIT prior to perform adhesion assays provides valuable insights into the behaviour of probiotics in the complex gut environment, contributing to the development of more effective probiotic formulations.

## 5 Conclusion

*Akkermansia muciniphila*, an intestinal symbiont that colonises the mucus layer, is considered a promising probiotic and it is currently approved for consumption in pasteurised form. Its use as viable cells is still being evaluated, even if numerous studies suggest that oral administration of *A. muciniphila* is safe. However, its dosage and potential side effects on human health remain unknown. Therefore, new studies are needed to clarify the characteristics of this bacterium. In our research, its ability to form aggregates in the gastric phase was highlighted. Thanks to this protective mechanism, *A. muciniphila* can maintain its viability until arrival in the gut, where its adhesion is much lower in comparison to the tested strain not subjected to simulated GIT. This evidence demonstrates the importance of conducting the two tests (simulated GIT and adhesion) sequentially and not separately, as this approach better simulates the physiological conditions in which the ingested microorganisms are found. In our opinion, this new knowledge may help to establish the right dose of ingestion of viable *A. muciniphila* cells, considering also that a major concern is the ability of this bacterium to adhere to intestinal cells and to degrade mucin, both of which appear to be limited by the stress encountered during GIT. This new knowledge may help to establish the right dose of ingestion of viable *A. muciniphila* cells, since the stress encountered during GIT limits the ability of this bacterium to adhere to intestinal cells and, as consequence, to degrade mucin.

## Data Availability

The raw data supporting the conclusions of this article will be made available by the authors, without undue reservation.
