# Supplementary material for: Akkermansia muciniphila: new insights into resistance to gastrointestinal stress, adhesion, and protein interaction with human mucins through optimised in vitro trials and bioinformatics tools
Source: Front Microbiol. 2024 Nov 5;15:1462220. doi: 10.3389/fmicb.2024.1462220 (PMC11573778; doi:10.3389/fmicb.2024.1462220)
Supplement: Supplementary file 1 [file Table_1.docx]

**Supplementary Table S1.** Comparison of adhesion percentages to CaCo2, HT-29 and HT29-MTX cell lines of *A. muciniphila* ATCC BAA-835 and *L. rhamnosus* GG ATCC 53103 tested at concentrations of 8, 7, 6, 5, 4, 3 and 2 Log CFU/mL.

|  | | **Adhesion (%) mean ± SD** | | **T-test** |
| --- | --- | --- | --- | --- |
| **Cell line** | **Bacterial concentration (Log CFU/mL)** | ***A. muciniphila* ATCC BAA-835** | ***L. rhamnosus* GG ATCC 53103** | **P-value** |
| **CaCo2** | 8 | 6.80 ± 0.185 | 5.84 ± 0.058 | 4.18E-06 |
|  | 7 | 10.20 ± 0.73 | 9.65 ± 0.26 | 1.50E-01 |
|  | 6 | 15.20 ± 0.202 | 10.9 ± 0.363 | 1.38E-06 |
|  | 5 | 114.00 ± 3.51 | 167.0 ± 6.30 | 2.16E-07 |
|  | 4 | 116.00 ± 3.30 | 170.0 ± 16.90 | 1.04E-04 |
|  | 3 | 438.00 ± 33.3 | 252.0 ± 6.04 | 1.80E-06 |
|  | 2 | 470.00 ± 21.6 | 261.0 ± 60.80 | 8.93E-05 |
| **HT-29** | 8 | 7.08 ± 0.172 | 5.86 ± 0.179 | 4.18E-06 |
|  | 7 | 10.40 ± 0.324 | 9.34 ± 0.485 | 3.20E-03 |
|  | 6 | 11.00 ± 0.536 | 10.91 ± 0.293 | 8.32E-01 |
|  | 5 | 74.30 ± 0.524 | 13.50 ± 1.66 | 7.83E-13 |
|  | 4 | 91.70 ± 0.61 | 13.10 ± 0.29 | 2.20E-16 |
|  | 3 | 92.80 ± 1.41 | 28.10 ± 0.599 | 1.76E-13 |
|  | 2 | 97.10 ± 5.79 | 30.10 ± 8.58 | 5.13E-07 |
| **HT29-MTX** | 8 | 6.82 ± 0.165 | 5.75 ± 0.082 | 1.17E-06 |
|  | 7 | 39.80 ± 0.581 | 12.10 ± 0.582 | 1.08E-12 |
|  | 6 | 66.60 ± 1.40 | 51.60 ± 0.538 | 1.70E-08 |
|  | 5 | 131.00 ± 0.822 | 58.10 ± 0.247 | 6.57E-16 |
|  | 4 | 455.00 ± 11.0 | 80.60 ± 2.37 | 1.19E-12 |
|  | 3 | 465.00 ± 7.91 | 79.00 ± 0.822 | 5.78E-14 |
|  | 2 | 482.00 ± 10.0 | 83.80 ± 2.60 | 3.74E-13 |
