# Supplementary material for: Akkermansia muciniphila: new insights into resistance to gastrointestinal stress, adhesion, and protein interaction with human mucins through optimised in vitro trials and bioinformatics tools
Source: Front Microbiol. 2024 Nov 5;15:1462220. doi: 10.3389/fmicb.2024.1462220 (PMC11573778; doi:10.3389/fmicb.2024.1462220)
Supplement: Supplementary file 2 [file Table_2.docx]

**Supplementary Table S2.** List of candidate mucinases of *A. muciniphila* (ATCC strain BAA-835/DSM 22959/JCM 33894/BCRC 81048/CCUG 64013/CIP 107961/Muc) searched in the Uniprot database.

| **Entry Name** | **Protein names** | **Gene Names** | **Amino acids** |
| --- | --- | --- | --- |
| B2UPI7_AKKM8 | Peptidase M60 domain-containing protein | Amuc_0627 | 506 |
| B2UQK5_AKKM8 | Peptidase M60 domain-containing protein | Amuc_0908 | 747 |
| B2ULE8_AKKM8 | Peptidase M60 domain-containing protein | Amuc_1514 | 660 |
| B2UR60_AKKM8 | Peptidase_M43 domain-containing protein | Amuc_1119 | 385 |
| B2UME7_AKKM8 | Peptidase M23 | Amuc_0253 | 289 |
| B2UNB8_AKKM8 | Peptidase M23 | Amuc_0391 | 225 |
| B2UNJ1_AKKM8 | Peptidase M23 | Amuc_0465 | 347 |
| B2UR47_AKKM8 | Peptidase M24 | Amuc_1106 | 427 |
| B2UM82_AKKM8 | Peptidase M28 | Amuc_0187 | 356 |
| B2ULX6_AKKM8 | Peptidase S1 and S6 chymotrypsin/Hap | Amuc_0176 | 334 |
| B2UMQ3_AKKM8 | Peptidase S1 and S6 chymotrypsin/Hap | Amuc_1791 | 254 |
| B2UL29_AKKM8 | Peptidase S11 D-alanyl-D-alanine carboxypeptidase 1 | Amuc_1480 | 323 |
| B2UN08_AKKM8 | Peptidase S15 | Amuc_1801 | 359 |
| B2UP79_AKKM8 | Oligopeptidase A (EC 3.4.24.70) | Amuc_2040 | 726 |
| B2UM07_AKKM8 | Carboxyl-terminal protease (EC 3.4.21.102) | Amuc_1631 | 748 |
| B2ULK5_AKKM8 | Peptidase M22 glycoprotease | Amuc_1571 | 222 |
| B2ULJ2_AKKM8 | Zinc metalloprotease (EC 3.4.24.-) | Amuc_1558 | 481 |
| B2UNU1_AKKM8 | Aspartyl protease | Amuc_0473 | 323 |
| B2UNU3_AKKM8 | Methyltransferase | Amuc_0475 | 191 |
| B2UP74_AKKM8 | ULP_PROTEASE domain-containing protein | Amuc_2035 | 251 |
| B2UPX2_AKKM8 | Trypsin-like protein serine protease typically periplasmic contain C-terminal PDZ domain-like protein | Amuc_0670 | 526 |
| B2UQG2_AKKM8 | Sulfatase | Amuc_0864 | 572 |
| B2UQQ0_AKKM8 | Sulfatase | Amuc_0953 | 1414 |
| B2UQX4_AKKM8 | Sulfatase | Amuc_1033 | 581 |
| B2UR15_AKKM8 | Sulfatase | Amuc_1074 | 552 |
| B2UR59_AKKM8 | Sulfatase | Amuc_1118 | 628 |
| B2URC2_AKKM8 | Sulfatase | Amuc_1182 | 465 |
| B2ULS2_AKKM8 | Sulfatase | Amuc_0121 | 526 |
| B2UM30_AKKM8 | Sulfatase | Amuc_1655 | 554 |
| B2UML7_AKKM8 | Sulfatase | Amuc_1755 | 562 |
| B2UNH7_AKKM8 | Sulfatase | Amuc_0451 | 719 |
| B2UNV9_AKKM8 | Sulfatase | Amuc_0491 | 500 |
| B2UPC5_AKKM8 | Sulfatase | Amuc_0565 | 542 |
| B2UPP3_AKKM8 | Formylglycine-generating enzyme, required for sulfatase activity, contains SUMF1/FGE domain | Amuc_2112 | 969 |
| B2ULI1_AKKM8 | Sialidase domain-containing protein | Amuc_1547 | 595 |
| B2UN42_AKKM8 | Exo-alpha-sialidase (EC 3.2.1.18) | Amuc_1835 | 674 |
| B2UPI5_AKKM8 | Exo-alpha-sialidase (EC 3.2.1.18) | Amuc_0625 | 419 |
| B2UPI3_AKKM8 | Glycosyl hydrolase BNR repeat-containing protein | Amuc_0623 | 436 |
| G1091_AKKM8 | Glycosyl hydrolase family 109 protein 1 (EC 3.2.1.-) | Amuc_0017 | 481 |
| G1092_AKKM8 | Glycosyl hydrolase family 109 protein 2 (EC 3.2.1.-) | Amuc_0920 | 473 |
| B2UKY7_AKKM8 | Glycosyl hydrolase family 98 putative carbohydrate binding module | Amuc_1438 | 639 |
| B2UQG1_AKKM8 | Glycosyl hydrolase family 88 | Amuc_0863 | 744 |
| B2UPN9_AKKM8 | Glycoside hydrolase family 16 | Amuc_2108 | 319 |
| B2UMS6_AKKM8 | Glycoside hydrolase family 2 sugar binding | Amuc_0290 | 986 |
| B2UQH3_AKKM8 | Glycoside hydrolase family 16 | Amuc_0875 | 313 |
| B2UM81_AKKM8 | Glycoside hydrolase family 95 | Amuc_0186 | 788 |
| B2UR61_AKKM8 | Glycosyl hydrolase | Amuc_1120 | 796 |
| B2UPU3_AKKM8 | Glycoside hydrolase family 18 | Amuc_2164 | 1233 |
| B2UNV0_AKKM8 | Hydrolase of the alpha/beta superfamily | Amuc_0482 | 272 |
| B2UQU5_AKKM8 | Hydrolase of the alpha/beta superfamily | Amuc_1003 | 377 |
| B2ULB7_AKKM8 | Alpha-N-acetylglucosaminidase (EC 3.2.1.50) | Amuc_0060 | 848 |
| B2URG0_AKKM8 | Alpha-N-acetylglucosaminidase (EC 3.2.1.50) | Amuc_1220 | 852 |
| B2UPZ9_AKKM8 | Beta-glucanase | Amuc_0697 | 381 |
| B2UQ00_AKKM8 | Beta-glucanase | Amuc_0698 | 369 |
| GLAA_AKKM8 | Alpha-1,3-galactosidase A (EC 3.2.1.n1) (Exo-alpha-galactosidase A) (EC 3.2.1.22) | Amuc_1463 | 596 |
| GLAB_AKKM8 | Alpha-1,3-galactosidase B (EC 3.2.1.n1) (EC 3.2.1.n2) (Exo-alpha-galactosidase B) (EC 3.2.1.22) | Amuc_0480 | 794 |
| B2UQF3_AKKM8 | Alpha-galactosidase (EC 3.2.1.22) | Amuc_0855 | 716 |
| B2URC7_AKKM8 | Alpha-galactosidase (EC 3.2.1.22) (Melibiase) | Amuc_1187 | 533 |
| B2UM40_AKKM8 | Beta-galactosidase (EC 3.2.1.23) (Lactase) | Amuc_1666 | 1289 |
| B2UM60_AKKM8 | Beta-galactosidase (EC 3.2.1.23) | Amuc_1686 | 780 |
| B2UQ71_AKKM8 | Beta-galactosidase (EC 3.2.1.23) | Amuc_0771 | 643 |
| B2UQ26_AKKM8 | Glucan endo-1,3-beta-D-glucosidase (EC 3.2.1.39) | Amuc_0724 | 290 |
| B2UQC2_AKKM8 | beta-galactosidase (EC 3.2.1.23) (Lactase) | Amuc_0824 | 1264 |
| B2UL68_AKKM8 | Alpha-L-fucosidase (EC 3.2.1.51) | Amuc_0010 | 538 |
| B2ULU6_AKKM8 | Alpha-L-fucosidase (EC 3.2.1.51) | Amuc_0146 | 711 |
| B2UNB9_AKKM8 | Coagulation factor 5/8 type domain protein | Amuc_0392 | 709 |
| B2UQE4_AKKM8 | Coagulation factor 5/8 type domain protein | Amuc_0846 | 704 |
| H2018_AKKM8 | Beta-hexosaminidase Amuc_2018 (EC 3.2.1.52) (Beta-N-acetylhexosaminidase Am2301) | Amuc_2018 | 493 |
| H2136_AKKM8 | Beta-hexosaminidase Amuc_2136 (EC 3.2.1.52) (Beta-N-acetylhexosaminidase Am2136) | Amuc_2136 | 756 |
| H0868_AKKM8 | Beta-hexosaminidase Amuc_0868 (EC 3.2.1.52) (Beta-N-acetylhexosaminidase Am0868) | Amuc_0868 | 549 |
| B2UM43_AKKM8 | Beta-N-acetylhexosaminidase (EC 3.2.1.52) | Amuc_1669 | 547 |
| B2UN02_AKKM8 | Beta-N-acetylhexosaminidase (EC 3.2.1.52) | Amuc_0369 | 665 |
| B2UN22_AKKM8 | Beta-N-acetylhexosaminidase (EC 3.2.1.52) | Amuc_1815 | 728 |
| B2UNC4_AKKM8 | Beta-N-acetylhexosaminidase (EC 3.2.1.52) | Amuc_0397 | 722 |
| B2UNM1_AKKM8 | Beta-N-acetylhexosaminidase (EC 3.2.1.52) | Amuc_1924 | 664 |
| B2UP58_AKKM8 | Beta-N-acetylhexosaminidase (EC 3.2.1.52) | Amuc_2019 | 504 |
| B2UPP0_AKKM8 | Beta-N-acetylhexosaminidase (EC 3.2.1.52) | Amuc_2109 | 353 |
| B2UPS8_AKKM8 | Beta-N-acetylhexosaminidase (EC 3.2.1.52) | Amuc_2148 | 549 |
| B2UQX3_AKKM8 | Beta-N-acetylhexosaminidase (EC 3.2.1.52) | Amuc_1032 | 518 |
| B2UPX9_AKKM8 | Beta-hexosaminidase | Amuc_0677 | 159 |
| B2UPX2_AKKM8 | Trypsin-like protein serine protease typically periplasmic contain C-terminal PDZ domain-like protein | Amuc_0670 | 526 |
