# Supplementary material for: Akkermansia muciniphila: new insights into resistance to gastrointestinal stress, adhesion, and protein interaction with human mucins through optimised in vitro trials and bioinformatics tools
Source: Front Microbiol. 2024 Nov 5;15:1462220. doi: 10.3389/fmicb.2024.1462220 (PMC11573778; doi:10.3389/fmicb.2024.1462220)
Supplement: Supplementary file 3 [file Supplementary_file_1.docx]

**Figure S1**. Survival of *A. muciniphila* ATCC BAA-835 to simulated GIT as ascertained with plate count (PC, Panels A and B) and Live/Dead assay (LD, Panels C and D). Panels A and C: counts performed on the initial microbial load (t0), after simulated passage in the mouth (Saliva) and after simulated passage in the stomach under different conditions (pH 2 and pH 3 for 30 and 90 min). Panels B and D: counts performed after a simulated passage in the Gut on bacterial cells previously exposed to pH 2 and pH 3 for 30 and 90 minutes. In each panel, Kruskal-Wallis test with Dunn’s test was used to evaluate significant differences (P-value ≤ 0.05). Different letters indicate significant differences calculated on 3 replicates.
