# Supplementary material for: Akkermansia muciniphila: new insights into resistance to gastrointestinal stress, adhesion, and protein interaction with human mucins through optimised in vitro trials and bioinformatics tools
Source: Front Microbiol. 2024 Nov 5;15:1462220. doi: 10.3389/fmicb.2024.1462220 (PMC11573778; doi:10.3389/fmicb.2024.1462220)
Supplement: Supplementary file 5 [file Supplementary_file_3.docx]

**Figure S3**. Viability of CaCo2, HT-29 and HT29-MTX cells measured as Neutral Red uptake after 24h of incubation with different concentrations of *Lbs. rhamnosus* GG (A) and *A. muciniphila* (B). ns, non-significant difference (Kruskal-Wallis test, P-values ≥ 0.05).
